# Potassium Starvation Limits Soybean Growth More than the Photosynthetic Processes across CO_2_ Levels

**DOI:** 10.3389/fpls.2017.00991

**Published:** 2017-06-08

**Authors:** Shardendu K. Singh, Vangimalla R. Reddy

**Affiliations:** ^1^Crop Systems and Global Change Laboratory, United States Department of Agriculture—Agricultural Research ServiceBeltsville, MD, United States; ^2^Wye Research and Education Center, University of Maryland, College ParkCollege Park, MD, United States

**Keywords:** chlorophyll fluorescence, *Glycine max*, nutrient utilization/uptake efficiency, root/shoot ratio, tissue potassium

## Abstract

Elevated carbon dioxide (eCO_2_) often enhances plant photosynthesis, growth, and productivity. However, under nutrient-limited conditions the beneficial effects of high CO_2_ are often diminished. To evaluate the combined effects of potassium (K) deficiency and eCO_2_ on soybean photosynthesis, growth, biomass partitioning, and yields, plants were grown under controlled environment conditions with an adequate (control, 5.0 mM) and two deficient (0.50 and 0.02 mM) levels of K under ambient CO_2_ (aCO_2_; 400 μmol mol^−1^) and eCO_2_ (800 μmol mol^−1^). Results showed that K deficiency limited soybean growth traits more than photosynthetic processes. An ~54% reduction in leaf K concentration under 0.5 mM K vs. the control caused about 45% less leaf area, biomass, and yield without decreasing photosynthetic rate (P_net_). In fact, the steady photochemical quenching, efficiency, and quantum yield of photosystem II, chlorophyll concentration (TChl), and stomatal conductance under 0.5 mM K supported the stable P_net_. Biomass decline was primarily attributed to the reduced plant size and leaf area, and decreased pod numbers and seed yield in K-deficient plants. Under severe K deficiency (0.02 mM K), photosynthetic processes declined concomitantly with growth and productivity. Increased specific leaf weight, biomass partitioning to the leaves, decreased photochemical quenching and TChl, and smaller plant size to reduce the nutrient demands appeared to be the means by which plants adjusted to the severe K starvation. Increased K utilization efficiency indicated the ability of K-deficient plants to better utilize the tissue-available K for biomass accumulation, except under severe K starvation. The enhancement of soybean growth by eCO_2_ was dependent on the levels of K, leading to a K × CO_2_ interaction for traits such as leaf area, biomass, and yield. A lack of eCO_2_-mediated growth and photosynthesis stimulation under severe K deficiency underscored the importance of optimum K fertilization for maximum crop productivity under eCO_2_. Thus, eCO_2_ compensated, at least partially, for the reduced soybean growth and seed yield under 0.5 mM K supply, but severe K deficiency completely suppressed the eCO_2_-enhanced seed yield.

## Introduction

Potassium (K) is the second most abundant element, after nitrogen (N), taken-up by plant roots. A large area of cropland is K deficient, partly due to the removal of native K by plants over years of cropping and an inadequate K application as compared with N and phosphorus (P) (Smil, 1999; Römheld and Kirkby, 2010). K deficiency limits crop growth and yield by adversely affecting vital plants processes, such as water relations and cellular turgidity, cell expansion, assimilate transport, and enzyme activation (Marschner, 1986; Pettigrew, 2008; Römheld and Kirkby, 2010). The atmospheric carbon dioxide (CO_2_) concentration is expected to double from the current level of 400 μmol mol^−1^ by the year 2100 (IPCC, 2013). An increase in CO_2_ concentration increases plant photosynthesis, growth, and yield, especially in C_3_ crops such as soybean. However, plant response to elevated CO_2_ (eCO_2_) depends on other environmental factors. For instance, soil-related constraints such as insufficient supply of mineral nutrients may limit the growth enhancement effects of eCO_2_ (Hoosbeek et al., 2002; Reddy and Zhao, 2005; Lenka and Lal, 2012). Moreover, an increased crop demand for mineral nutrients due to the use of high yielding cultivars and greater plant growth under eCO_2_ might exacerbate the limitations, especially under nutrient deficient conditions (Conroy, 1992; Rogers et al., 1993; Singh et al., 2015). K deficiency and eCO_2_ are expected to co-occur under natural conditions and their interaction may alter the degree of plant growth and physiological responses. Thus, the interaction study would help to elucidate whether the eCO_2_ compensates the adverse effects of K deficiency and the extent of changes in the response of plant growth and physiological attributes.

Crops including soybean are highly sensitive to K deficiency, with a strong impact on biomass accumulation, yield, and quality (Sale and Campbell, 1986; Pettigrew, 2008; Gerardeaux et al., 2009; Ma et al., 2013). K starvation decreases leaf K concentration and may suppress vital plant physiological processes without a substantial reduction in the rate of photosynthesis and before visible leaf symptoms occur (Bednarz and Oosterhuis, 1999; Gerardeaux et al., 2009; Kanai et al., 2011). Previous studies in cotton suggested a considerable decrease in leaf area and dry matter production without a marked reduction in leaf- or ground-based canopy photosynthesis under moderate K deficiency (Reddy and Zhao, 2005; Gerardeaux et al., 2009). Therefore, unlike N or P, K deficiency appears to limit plant growth and root development by suppressing the processes of supply and transport of sugars, metabolites, and other minerals among plant organs more than direct inhibition of carbon assimilation (Marschner, 1986; Cakmak et al., 1994; Reddy and Zhao, 2005; Gerardeaux et al., 2009; Römheld and Kirkby, 2010; Kanai et al., 2011; Singh and Reddy, 2016). However, several functional and structural limitations to the photosynthetic potential may occur under severe K deficiency. Prominent functional limitations include restricted diffusion of CO_2_ into and through leaves (stomatal or mesophyll conductance), diminished carboxylation capacity, chlorophyll biosynthesis, and assimilate transport. Structural limitations are attributed to the alterations in leaf morphology, size and density of stomata, and anatomical features such as leaf thickness and density, orientation of the chloroplasts, and the intercellular air space (Zhao et al., 2001; Gerardeaux et al., 2009; Jin et al., 2011; Battie-Laclau et al., 2014; Lu et al., 2016a).

Nutrient deficiency also affects biomass partitioning and nutrient allocation among plant parts (Gill et al., 1997; Reddy and Zhao, 2005; Singh et al., 2014). Since translocation mechanisms involving phloem loading and transport are interrupted by low tissue K concentration, this may lead to the impairment of partitioning and utilization of carbon reserves (Cakmak et al., 1994; Bednarz and Oosterhuis, 1999; Cakmak, 2005). A minimum concentration of tissue K is therefore essential to support metabolic processes, and uptake and transport mechanisms, and to maintain cellular turgidity (Siddiqi and Glass, 1981; Cakmak, 2005; Römheld and Kirkby, 2010). Gill et al. (1997) found increased K utilization efficiency (KUE) under moderate K stress in wheat genotypes. However, under nutrient-limited conditions, greater nutrient use efficiency has not always been observed (Singh and Reddy, 2014).

Although, CO_2_ enrichment stimulates plant photosynthesis and growth, K starvation may limit this stimulation (Hoosbeek et al., 2002; Reddy and Zhao, 2005). Nonetheless, a positive effect of elevated CO_2_ on plant growth is still expected under K deficiency due to an increased carbohydrate supply, which may aid the energy demands of the stressed plants (Ahmed et al., 1993; Singh et al., 2013b). Moreover, the growth and seed filling processes of soybean depend on the remobilization of resources such as carbohydrates, minerals, and amino acids among plant organs, and plant photosynthetic capacity. All of these processes are influenced by K nutrition and elevated CO_2_, which in combination may alter growth and physiological processes (Conroy, 1992; Reddy and Zhao, 2005; Singh and Reddy, 2016). Therefore, the interaction between K deficiency and eCO_2_ can modify the overall crop response to K deficiency in soybean. Studies evaluating the whole-plant response to the combination of K deficiency and elevated CO_2_ in soybean are extremely limited. We hypothesized that growth and photosynthetic responses to K starvation would differ in soybean depending on the level of K supplied, and that eCO_2_ would compensate, at least partially, for the adverse effect of K starvation on plant growth. The objectives of this study were to investigate the interactive effects of K and CO_2_ levels on soybean photosynthesis, growth, biomass partitioning, and nutrient allocation and utilization efficiencies.

## Materials and methods

### Experimental conditions

The experiment was conducted at the USDA-ARS facility in Beltsville, MD, USA, using six controlled environment growth chambers (EGC Corp., Chagrin Falls, OH, USA) with three chambers per CO_2_ level in 2013. The experiment was repeated over time using the same six chambers. Soybean (cv. Spencer) seeds were planted in 18 pots (volume 7.6 L, five seeds per pot) filled with quartz silica sand (#2Q-ROK®, US Silica Company, MD, USA) in each chamber. After emergence (4 days after planting, DAP), the treatments were initiated using a combination of two levels of CO_2_, 400 μmol mol^−1^ (ambient; aCO_2_) and 800 μmol mol^−1^ (elevated; eCO_2_), and three levels of K (potassium nitrate; KNO_3_), 5.00 mM (control), 0.50 mM, and 0.02 mM K (K deficiency), in a modified Hoagland's nutrient solution (Hewitt, 1952). The N concentration of the nutrient solution was adjusted using ammonium nitrate (NH_4_NO_3_). The nutrient solution was applied using an automated and computer-controlled drip system until pots started to flush from the bottom holes, 4–6 times during the day. Pots were flushed weekly with deionized water. Pots were thinned to one plant per pot 5 days after emergence. Pots were repositioned periodically within each chamber to minimize the effects of within-chamber heterogeneity. The CO_2_ treatments were re-randomized when the experiment was repeated to minimize potential chamber effects.

A 28/22°C day/night (12h/12h) air temperature was maintained to within ± 0.15°C in the growth chambers during the experiment. Light, as photosynthetically active radiation (PAR) of 900 μmol m^−2^ s^−1^ at plant canopy height, was supplied following emergence during the day using a combination of metal halide and high-pressure sodium lamps. The PAR at the plant canopy was monitored using a LI-COR Quantum Sensor connected to a LI-1000 data logger (LI-COR Inc., Lincoln, NE, USA) at multiple locations within each chamber on alternate days and adjusted to the mean value of 900 ± 15 μmol m^−2^ s^−1^ using controllable ballasts (Osram Sylvania, MA, USA). The daytime temperature and light were initiated at 6:00 h. Injection of either CO_2_ or CO_2_-free air was determined using a TC-2 controller that monitored CO_2_ every 3 s, measured from an absolute infrared gas analyzer (WMA-4PP-systems, Haverhill, MA, USA). The relative humidity was not controlled and varied between 50 and 70% among chambers.

### Growth measurements

Six pots per treatment (total 36 pots: 6 pots × 3 K levels × 2 CO_2_) were removed at each of 28 (full bloom), 42 (pod development), and 112 (maturity) DAP for three destructive plant harvests. Since the experiment was repeated, 72 pots (36 × 2) were destructively harvested at each of the three stages. Plant height and mainstem node numbers were recorded from each pot. Plants were separated into leaves, roots, stems, and pods. Since some of the leaves were dried at maturity, total leaf area (TLA) was measured only for the harvests conducted at 28 and 42 DAP, and to measure the specific leaf weight [SLW; mg dry weight (cm^2^ leaf area)^−1^]. Roots were washed in clean water. All plant parts were dried to constant weight at 70°C to determine the dry matter (DM). The seed and shell mass was determined from pods obtained at maturity after drying at 35°C in forced-ventilation air for 10 days.

### Measurements of photosynthetic parameters

Photosynthetic parameters were measured several times between 24 and 72 DAP on the uppermost fully expanded leaves from the stem apex in 3–4 plants per treatment between 9:00 and 14:00 h using a LI-6400XT portable photosynthesis system (LI-COR Inc.) with an integrated fluorescence chamber head (LI-COR 6400-40 Leaf Chamber Fluorometer). These measurements were made at a PAR of 1500 μmol photons m^−2^ s^−1^; the leaf temperature of the instrument was set to 28°C; the CO_2_ concentration was set to match the CO_2_ treatments; and the relative humidity inside the LI-COR leaf chamber varied between 45 and 60%. Gas exchange and chlorophyll fluorescence measurements were made simultaneously when a steady state (around 4–6 min) was obtained. The net photosynthesis rate (P_net_), stomatal conductance (*g*_s_), transpiration (T), sub-stomatal (C_*i*_) CO_2_ concentration, and photochemical yield of CO_2_ fixation (Φ_CO2_) were computed from the instrument's software. For the chlorophyll fluorescence measurements, the steady-state chlorophyll fluorescence (F_*s*_) was measured first followed by maximal fluorescence (${\text{F}}_{\text{m}}^{\prime }$) by providing a 0.8 s flash of saturating light of >8,000 μmol m^−2^ s^−1^ PAR. Immediately following, the minimal fluorescence of the light-adapted leaf (${\text{F}}_{o}^{\prime }$) was obtained by providing a dark flash by turning off the actinic light briefly while using far-red LEDs (centered wavelength 740 nm). The far-red radiation drives Photosystem I (PSI) to help drain Photosystem II (PSII) electrons. The variable fluorescence yield in the light-adapted leaves (${\text{F}}_{v}^{\prime }$), efficiency of energy harvesting by oxidized (open) PSII reaction centers in light (${\text{F}}_{v}^{\prime }$/${\text{F}}_{m}^{\prime }$), proportion of the PSII unit in open state or photochemical quenching (q_P_), and photochemical quantum yield of PSII electron transport rate (Φ_PSII_) were determined following Genty et al. (1989) using the equations:

(1) $${\text{F}}_{\text{v}}^{\prime }={\text{F}}_{\text{m}}^{\prime }-{\text{F}}_{\text{o}}^{\prime }$$

(2) $${\text{F}}_{\text{v}}^{\prime }/{\text{F}}_{\text{m}}^{\prime }=({\text{F}}_{\text{m}}^{\prime }-{\text{F}}_{\text{o}}^{\prime })/{\text{F}}_{\text{m}}^{\prime }$$

(3) $${\text{q}}_{\text{P}}=({\text{F}}_{\text{m}}^{\prime }-{\text{F}}_{\text{s}})/({\text{F}}_{\text{m}}^{\prime }-{\text{F}}_{\text{o}}^{\prime })$$

(4) $$\Phi_{\text{PSII}}\;=({\text{F}}_{\text{m}}^{\prime }-{\text{F}}_{\text{s}})/{\text{F}}_{\text{m}}^{\prime }$$

Details of the fluorescence measurements and other operational details are available in the instrument's manual (LI-6400 Instruction Manual, version 5, LI-COR Inc., Lincoln, Nebraska, USA).

### Tissue chlorophyll, potassium, and nitrogen measurements

The total chlorophyll concentration (TChl), and leaf K and N concentrations were determined from the uppermost fully expanded leaves that were used for gas exchange measurements. The leaves were detached immediately after the gas exchange measurements and fresh and dry weights were determined after drying to a constant weight at 70°C. The K and N concentrations were also determined in each plant organ (i.e., leaf, stem, root, pod, shell, and seed) at all harvests. The weighted whole-plant (referred to as plant) K and N concentrations were estimated as the sum of the products of the dry mass of plant organs and their nutrient concentration divided by the total biomass (Singh et al., 2015). Similarly, the weighted pod K and N concentrations at the final harvest were estimated as the sum of the products of the dry mass of seeds and shells and their nutrient concentrations divided by pod dry weight. The K and N utilization efficiencies for biomass production (KUE and NUE, respectively) were estimated by dividing total biomass by the whole-plant K or N concentration (Singh et al., 2015). The total K or N absorbed (mg plant^−1^) per unit of root biomass was used as an indicator of K or N uptake efficiency (KUpE and NUpE, respectively).

Chlorophyll was extracted by placing two 0.95 cm^2^ leaf disks for each leaf in a vial containing 7 mL of dimethyl sulfoxide and incubating in the dark for 24 h. Thereafter, the absorbance of the supernatant was measured at 664 and 648 nm by using a UV-2101-PC spectrophotometer (Shimadzu Corp., Columbia MD, USA). The total chlorophyll was estimated by using the equation of Lichtenthaler (1987) and expressed on a leaf area basis (μg cm^−2^). The dry matter of plant parts was ground using a Wiley Mill (Wiley® Mill, Thomas Scientific, NJ, USA) to pass through a 1 mm screen. The K concentration was determined in the ground dried-tissue in the Agriculture Diagnostic Laboratory, University of Arkansas, AR, USA, using a standard procedure (Plank, 1992). In brief, 0.25 g tissue was digested at 110–120°C for ~2 h using concentrated HNO_3_ and hydrogen peroxide on an Al digestion block in calibrated 50 ml tubes. The samples were then brought to a 25 mL total volume with deionized water. The digestates were analyzed by using a Spectro ARCOS EOP-Inductively Coupled Plasma Spectrophotometer (Spectro Analytical Instruments, Mahwah, NJ, USA). The N concentration of the plant tissues was determined by combustion using a CHN-2000 (Carbon Hydrogen Nitrogen-2000, LECO Corporation, St. Joseph, MI, USA).

### Statistical analysis

**Figure 1:**
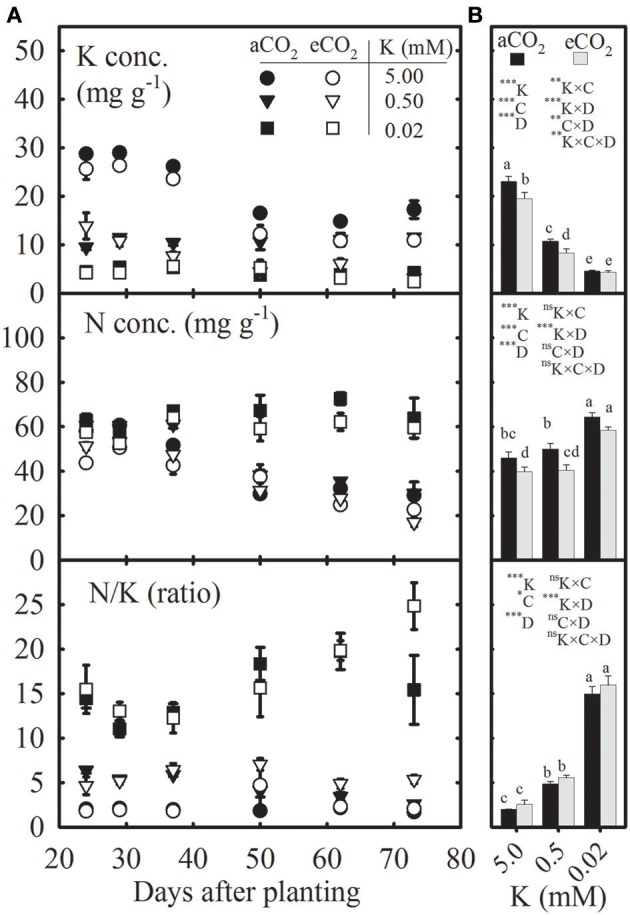
Seasonal variation **(A)** and mean values **(B)** of potassium (K) and nitrogen (N) concentrations and N/K ratio in uppermost fully expanded soybean leaves as affected by K treatments under ambient (aCO_2_; filled symbols/black bars, 400 μmol mol^−1^) and elevated CO_2_ (eCO_2_; open symbols/gray bars, 800 μmol mol^−1^). Symbols **(A)** represent the mean ± SE of 6–8 individuals measured across the repeated experiment between 24 and 72 days after planting (DAP). Bars **(B)** represent seasonal means + SE, and the analysis of variance for the effects of K, CO_2_ (C), DAP (D), and their interactions is also shown with the significance levels, where ^*^, ^**^, ^***^, and ^ns^ represent *P* ≤ 0.05, *P* ≤ 0.01, *P* ≤ 0.001, and *P* > 0.05, respectively. Bars with different letters are significantly different at α = 0.05. Error bars smaller than the symbols are not visible.

**Figure 2:**
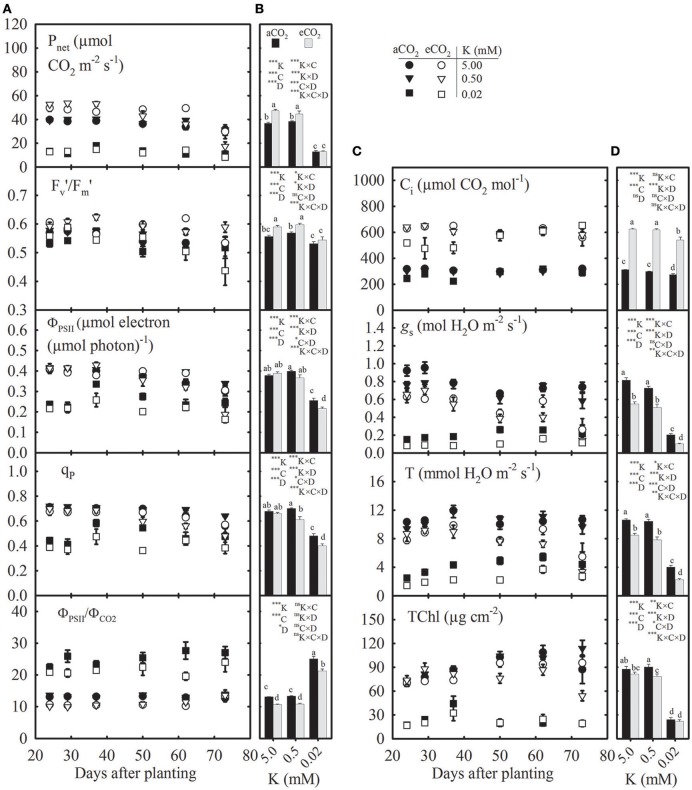
Seasonal variation **(A,C)** and mean values **(B,D)** of photosynthesis (P_net_), efficiency of energy harvesting by oxidized (open) photosystem II (PSII) reaction centers in light (${\text{F}}_{\text{v}}^{\prime }$/${\text{F}}_{\text{m}}^{\prime }$), photochemical quantum yield of PSII electron transport rate (Φ_PSII_), proportion of the PSII unit in open state or photochemical quenching (q_P_), ratio between Φ_PSII_ and quantum yield of CO_2_ fixation (Φ_PSII_/Φ_CO2_), sub-stomatal CO_2_ concentration (C_i_), stomatal conductance (*g*_s_), transpiration (T), and total chlorophyll concentration (TChl) of the uppermost fully expanded soybean leaves as affected by K supply under ambient (aCO_2_; filled symbols/black bars, 400 μmol mol^−1^) and elevated CO_2_ (eCO_2_; open symbols/gray bars, 800 μmol mol^−1^). See Figure 1 for other information.

Statistical analyses were performed using SAS software (SAS Enterprise Guide, 4.2, SAS Institute Inc., NC, USA). To test for the effect of treatments and their interaction, the SAS procedure PROC MIXED with Kenward-Rogers (kr) adjustment of degrees of freedom was used for analysis of variance (ANOVA) using the individual measurements from each repetition of the experiment. Treatments (K and CO_2_) and their interaction were considered as fixed effects and repetition of the experiment as a random effect. Treatment comparisons were conducted by using the least square means (LSMEANS) procedure (at α = 0.05) when the main effect and/or the interaction was significant at *P* ≤ 0.05, and the letter grouping was obtained using the pdmix800 macro (Saxton, 1998). Since the plant age (DAP) was likely to have a significant impact on variables measured throughout the season (Figures 1, 2), the DAP and its interactions with the treatments were included as fixed effects in the analysis. However, the treatment comparison was conducted only on the seasonal means (averaged across DAP) for each variable. PROC REG, PROC GLM, or PROC NLIN procedures of SAS was used for linear, polynomial, or non-linear regression analyses to calculate the coefficients and to establish the relationships between the plant attributes.

## Results

There were no visual K deficiency symptoms observed on the soybean leaves of control plants. The leaves of plants grown under 0.5 mM K treatment showed some marginal chlorosis on the tips and edges of a few leaves in the top half of the plant canopy at around 58 DAP, especially at eCO_2_. Under severe K (0.02 mM K) deficiency, stems were slender, and the lower leaves began to show chlorosis around the tips and edges by 20 DAP. At the advanced stage, these symptoms extended inward toward the midrib and interveinal chlorosis/necrosis and some degree of inward curling of leaves was observed as plants aged under both CO_2_ treatments. All leaves of plants grown under severe K deficiency showed various stages of K deficiency symptoms during the later growth stages. Similar K deficiency symptoms have been reported in the leaves of soybean and cotton plants (Bell et al., 1987; Bednarz et al., 1998).

### K and N concentrations in the uppermost fully expanded leaves decreased or increased, respectively, under K starvation, but tended to decline at eCO_2_

The K and N concentrations in the uppermost fully expanded leaves tended to decline with plant age (DAP), especially under the control treatment (Figure 1A). However, the N concentration under severe K deficiency (0.02 mM K) increased at ~50 DAP. Compared with the control, the seasonal mean (averaged across the DAP) K concentration significantly declined by 54–79%, but the mean N concentration increased by 7–45% under K starvation (Figure 1B). The seasonal means of leaf K and N concentrations were 7.35–20.03% lower under eCO_2_ vs. aCO_2_ across K treatments. The N/K ratio was ~2- to 8-fold greater under K deficiency regardless of CO_2_ level throughout the growing season, which was also reflected by the seasonal mean data (Figures 1A,B).

### K and N concentration of plant organs decreased or increased, respectively, under K starvation, but responses to eCO_2_ varied

K deficiency significantly decreased the tissue K concentration by over 50% in all plant organs as compared with the control (Table 1). In contrast, K deficiency caused an accumulation of N in all plant organs with the highest increase observed for shells (>250%) followed by the leaves and stems (>150%) at maturity. The accumulation of tissue N concentration was relatively greater under 0.02 vs. 0.5 mM K treatment. In the vegetative plant parts, on average, stems had the greatest K concentration but leaves had the greatest N concentration. In the reproductive parts, seeds or pods had the highest concentration of both K and N. The eCO_2_ tended to decrease tissue K concentration of plants, leaves, and pods at maturity, particularly under the control and 0.5 mM K. A significant effect of CO_2_ treatment on tissue N concentration was mainly observed 28 and 42 DAP in plants, leaves, and roots. The K × CO_2_ interaction was also observed for K and N concentrations of plants, stems, and pods in at least one out of the three harvests and was attributed to the minimal or negative effects of CO_2_ under the severe K deficiency. For instance, the K concentration in stems at 28 DAP increased by 11–26% under eCO_2_ vs. aCO_2_ for the two high K treatments but decreased by 34% for the lowest (0.02 mM) K treatments.

**Table 1:** Effect of CO_2_ (μmol mol^−1^) and potassium (K; mM) treatments on K and nitrogen (N) concentration in tissues of soybean plants and organs 28, 42, and 112 (or maturity) days after planting (DAP).

**CO_2_**	**K**	**K (mg g^−1^)**							**N (mg g^−1^)**						
		**Plant**	**Leaves**	**Stems**	**Roots**	**Pods**	**Shells**	**Seeds**	**Plant**	**Leaves**	**Stems**	**Roots**	**Pods**	**Shells**	**Seeds**
**28 DAP**															
400	5.00	34.37^a^	26.62^a^	43.85^b^	41.51^a^				49.21^b^	62.23^ab^	34.07^d^	36.23^cd^			
	0.50	13.17^b^	12.51^b^	14.31^d^	13.40^b^				53.35^a^	60.69^ab^	44.80^bc^	48.38^a^			
	0.02	7.33^c^	5.56^c^	11.19^d^	8.67^bc^				55.55^a^	63.43^a^	45.53^b^	41.03^bc^			
800	5.00	35.52^a^	27.73^a^	48.91^a^	43.61^a^				42.97^c^	54.35^d^	32.49^d^	35.66^d^			
	0.50	14.85^b^	13.26^b^	18.06^c^	14.43^b^				50.05^b^	59.58^bc^	41.38^c^	41.61^b^			
	0.02	6.22^c^	5.80^c^	7.36^e^	6.11^c^				54.36^a^	57.43^c^	52.36^a^	43.41^ab^			
ANOVA^†^	K	<0.0001	<0.0001	<0.0001	<0.0001				<0.0001	0.1050	<0.0001	<0.0001			
	CO_2_	0.4197	0.1112	0.1231	0.9117				<0.0001	<0.0001	0.5554	0.2800			
	K × CO_2_	0.2413	0.7111	0.002	0.5103				0.3254	0.1722	0.0002	0.0515			
**42 DAP**															
400	5.00	28.8^a^	27.22^a^	31.05^b^	38.89^a^	29.12^a^			39.48^cd^	57.76^bc^	24.27^c^	29.84^b^	38.63^b^		
	0.50	9.24^b^	10.70^c^	7.91^c^	8.26^b^	14.52^b^			46.65^b^	59.37^b^	34.06^b^	42.69^a^	43.84^a^		
	0.02	4.97^c^	5.09^e^	4.90^e^	3.29^c^	16.70^b^			59.84^a^	68.39^a^	46.88^a^	45.28^a^	41.53^ab^		
800	5.00	29.73^a^	25.57^b^	34.22^a^	36.62^a^	29.56^a^			35.73^d^	52.07^d^	21.40^c^	25.99^b^	40.27^ab^		
	0.50	8.02^b^	9.38^d^	7.42^cd^	5.31^bc^	14.64^b^			40.91^c^	53.34^cd^	32.66^b^	31.68^b^	41.47^ab^		
	0.02	4.69^c^	4.49^e^	5.29*d*^e^	3.85^c^	17.36^b^			55.68^a^	60.80^b^	46.93^a^	43.69^a^	44.05^ab^		
ANOVA	K	<0.0001	<0.0001	<0.0001	<0.0001	<0.0001			<0.0001	<0.0001	<0.0001	<0.0001	0.0429		
	CO_2_	0.5785	0.0345	0.0846	0.0856	0.6421			0.0007	<0.0001	0.1898	0.0245	0.7031		
	K × CO_2_	0.4460	0.284	0.0758	0.2376	0.9632			0.7968	0.8577	0.5386	0.3276	0.2465		
**112 DAP**															
400	5.00	25.19^a^	24.38^a^	25.88^a^	6.33^b^	26.36^a^	36.65^a^	22.69^a^	37.72^d^	24.93^bc^	17.56^bc^	20.48^b^	48.26^d^	8.11^d^	62.83^d^
	0.50	14.05^c^	9.24^c^	11.48^b^	1.10^c^	17.38^b^	13.99^c^	18.81^ab^	42.51^c^	27.28^b^	20.51^b^	25.10^b^	54.46^bc^	12.93^c^	70.81^bc^
	0.02	4.88^e^	4.74^d^	2.36^c^	0.82^c^	9.11^d^	4.92^d^	13.39^c^	60.21^a^	66.87^a^	44.06^a^	36.37^a^	58.47^a^	40.98^a^	77.61^a^
800	5.00	23.42^b^	17.95^b^	26.62^a^	8.42^a^	24.89^a^	33.48^b^	21.79^a^	38.87^d^	21.85^c^	15.17^c^	24.87^b^	51.03^cd^	6.72^d^	67.16^c^
	0.50	9.64^d^	5.62^d^	6.51^b^	1.13^c^	12.34^c^	5.86^d^	14.93^bc^	45.49^c^	30.21^b^	18.51^bc^	26.77^b^	57.62^ab^	14.10^c^	74.19^ab^
	0.02	4.44^e^	4.26^d^	3.53^c^	1.46^c^	8.59^d^	5.25^d^	13.94^c^	55.41^b^	61.78^a^	43.87^a^	40.52^a^	50.85^d^	37.47^b^	72.17^b^
ANOVA	K	<0.0001	<0.0001	<0.0001	<.0001	<0.0001	<0.0001	<0.0001	<0.0001	<0.0001	<0.0001	<0.0001	<0.0001	<0.0001	<0.0001
	CO_2_	0.0351	0.0221	0.2532	0.1041	<0.0001	0.0002	0.0651	0.7875	0.2751	0.2222	0.0742	0.5778	0.188	0.4988
	K × CO_2_	0.0012	0.2351	0.8451	0.3079	0.0002	0.0017	0.1253	0.2315	0.1293	0.7376	0.7566	<0.0001	0.1332	0.0008

† *The significance test (P-values) of the analysis of variance (ANOVA) between K and CO_2_ are given. Within columns, means followed by same letters are not significantly different at α = 0.05*.

*Data are the mean of 12 individuals*.

### Photosynthetic parameters primarily declined at the lowest K supply but varied under eCO_2_ across K levels

The photosynthetic parameters appeared to be consistent in the growing season except at the beginning or end of the measurement period, which might have led to the significant effect of plant age (DAP) on these traits (Figure 2). Averaged across growth CO_2_, the seasonal mean of P_net_, ${\text{F}}_{\text{v}}^{\prime }$/${\text{F}}_{\text{m}}^{\prime }$, Φ_PSII_, q_P_, C_i_, *g*_s_, T, and TChl significantly declined by ≈70, 6, 38, 33, 12, 77, 66, and 75%, respectively, particularly at the lowest (0.02 mM K) vs. control K treatment (Figures 2B,D). However, the Φ_PSII_/Φ_CO2_ ratio increased by 94% under the same condition. The eCO_2_ significantly increased seasonal mean P_net_ (15–28%) under the two high K treatments but not under the lowest K treatment leading to a K × CO_2_ interaction. The eCO_2_ stimulated ${\text{F}}_{\text{v}}^{\prime }/{\text{F}}_{\text{m}}^{\prime }$ while it tended to decrease Φ_PSII_, q_P_, and Φ_PSII_/Φ_CO2_ (Figure 2B). The mean *g*_s_ and T consistently declined (20–51%) at eCO_2_ vs. aCO_2_ across K treatments (Figure 2D). The mean TChl also decreased by 10–20% under eCO_2_, particularly in the two high K treatments.

### Growth parameters consistently declined under K starvation and were enhanced by eCO_2_ at higher K levels

K starvation significantly decreased all growth parameters, except the mainstem node numbers, across the three harvests (Table 2). In contrast, eCO_2_ significantly stimulated TLA, DM production, pods, and seed yield at the two high K treatments (control and 0.5 mM K). However, this stimulation was either not observed or not significant at the lowest K (0.02 mM) treatment, leading to a K × CO_2_ interaction. Averaged across the CO_2_ levels, as compared with the control, the K deficiency caused ~45 and 95% decline under 0.5 and 0.02 mM K, respectively, for TLA, DM, and seed yield across all harvests (Tables 2, 3). The eCO_2_ stimulated TLA and DM by ≈50% and seed yield by ≈35%, when averaged between the two high K treatments. Moreover, these eCO_2_-mediated enhancements for TLA and DM were ≈30% greater under the 0.5 mM K than under the control treatment, whereas for the seed yield the enhancement was 14%. Averaged between CO_2_ levels and harvests, the plant C/N ratio was 10.3 under control K but decreased to 7.2 in severely K-deficient plants. Compared with the control K treatment, the pod and seed numbers were also decreased by 65–71% under K starvation but were increased under eCO_2_ by 20–49%, particularly at the two high K treatments (Table 2). The individual seed weight (g seed^−1^) also declined under severe K deficiency by ≈60% and was unaffected by growth CO_2_.

**Table 2:** Effect of CO_2_ (μmol mol^−1^) and potassium (K; mM) treatments on plant height (PH; cm), mainstem node numbers (NN; plant^−1^), total leaf area (TLA; cm^2^ plant^−1^), total dry matter (DM; g plant^−1^), plant C/N ratio, number of pods (Pod no.; plant^−1^), seed number (Seed no.; plant^−1^), and individual seed weight (g Seed^−1^) of soybean at 28, 42, and 112 (or maturity) days after planting (DAP).

**CO_2_**	**K**	**PH**	**NN**	**TLA**	**DM**	**C/N**	**Pod no**.	**Seed no**.	**g Seed^−1^**
**28 DAP**									
400	5.00	46.6^a^	8.92^a^	1098.2^b^	7.85^b^	8.38^b^			
	0.50	32.5^b^	8.83^a^	413.6^d^	3.82^c^	7.80^c^			
	0.02	23.3^c^	6.75^b^	98.8^e^	0.96^d^	7.40^d^			
800	5.00	44.7^a^	9.83^a^	1494.9^a^	11.06^a^	8.93^a^			
	0.50	35.5^b^	9.75^a^	665.5^c^	6.71^b^	8.27^b^			
	0.02	25.6^c^	7.00^b^	92.2^e^	0.99^d^	7.63^cd^			
ANOVA^†^	K	<0.0001	<0.0001	<0.0001	<0.0001	<0.0001			
	CO_2_	0.2658	0.0234	0.0001	<0.0001	<0.0001			
	K × CO_2_	0.1088	0.5783	0.0088	0.0012	0.3465			
**42 DAP**									
400	5.00	134.1^a^	15.5^c^	3973^b^	36.8^b^	10.3^b^	57.4^a^		
	0.50	112.8^b^	16.1^bc^	1774^d^	21.6^c^	9.0^c^	25.3^c^		
	0.02	75.3^c^	12.5^d^	303^e^	3.5^d^	7.0^d^	1.6^d^		
800	5.00	141.4^a^	16.8^ab^	5807^a^	51.4^a^	11.5^a^	61.6^a^		
	0.50	116.5^b^	17.3^a^	3229^c^	35.4^b^	10.5^b^	33.8^b^		
	0.02	74.6^c^	13.1^d^	315^e^	3.6^d^	7.6^d^	0.4^d^		
ANOVA	K	<0.0001	<0.0001	<0.0001	<0.0001	<0.0001	<0.0001		
	CO_2_	0.2692	0.0261	0.0061	<0.0001	0.0351	0.1139		
	K × CO_2_	0.5758	0.4801	0.0481	0.0004	0.2249	0.2607		
**112 DAP**									
400	5.00	155.4^b^	18.1^b^		206.5^b^	11.7^a^	163.7^b^	367.0^b^	0.244^a^
	0.50	143.1^b^	19.8^a^		99.1^d^	10.3^bc^	89.0^d^	177.5^d^	0.244^a^
	0.02	113.7^c^	19.1^ab^		18.9^e^	6.5^e^	16.5^e^	13.3^e^	0.104^b^
800	5.00	171.5^a^	19.2^ab^		254.9^a^	11.0^ab^	197.7^a^	460.7^a^	0.253^a^
	0.50	154.6^b^	20.3^a^		161.9^c^	10.0^c^	122.2^c^	264.7^c^	0.235^a^
	0.02	156.7^b^	20.3^a^		24.3^e^	7.4^d^	20.2^e^	11.2^e^	0.095^b^
ANOVA	K	<0.0001	0.0306		<0.0001	<0.0001	<0.0001	<0.0001	<0.0001
	CO_2_	<0.0001	0.0938		<0.0001	0.7669	0.0341	<0.0001	0.6437
	K × CO_2_	0.2438	0.8174		<0.0001	0.2803	0.0468	0.0241	0.3354

† *The significance test (P-values) of the analysis of variance (ANOVA) between K and CO_2_ are given. Within columns, means followed by same letters are not significantly different at α = 0.05*.

*See Table 1 for other information*.

**Table 3:** Effect of CO_2_ (μmol mol^−1^) and potassium (K; mM) on biomass partitioning of soybean plants at 28, 42, and 112 (maturity) days after planting (DAP).

**CO_2_**	**K**	**Tissue Biomass (g plant^−1^)**						**Fraction of total biomass (%)**					
		**Leaves**	**Stems**	**Roots**	**Pods**	**Shells**	**Seeds**	**Leaves**	**Stems**	**Roots**	**Pods**	**Shells**	**Seeds**
**28 DAP**													
400	5.00	4.10^b^	2.31^b^	1.43^b^				52.25^b^	29.50^a^	18.25^bc^			
	0.50	2.07^c^	0.99^d^	0.76^c^				54.31^b^	25.51^bc^	20.18^ab^			
	0.02	0.58^d^	0.23^e^	0.15^d^				60.60^a^	23.58^c^	15.82^cd^			
800	5.00	5.84^a^	3.10^a^	2.13^a^				52.81^b^	27.96^ab^	19.24^ab^			
	0.50	3.43^b^	1.81^c^	1.48^b^				51.33^b^	26.87^ab^	21.80^a^			
	0.02	0.61^d^	0.24^e^	0.14^d^				62.57^a^	23.51^c^	13.92^d^			
ANOVA^†^	K	<0.0001	<0.0001	<0.0001				<0.0001	<0.0001	<0.0001			
	CO_2_	<0.0001	<0.0001	<0.0001				0.8992	0.9178	0.7790			
	K × CO_2_	0.0023	0.004	0.0023				0.2246	0.3589	0.196			
**42 DAP**													
400	5.00	16.62^b^	13.12^b^	4.24^b^	2.83^a^			45.21^c^	35.52^b^	11.54^a^	7.73^a^		
	0.50	10.68^c^	6.55^c^	2.52^c^	1.87^b^			49.46^b^	30.27^c^	11.73^a^	8.54^a^		
	0.02	2.07^d^	0.96^d^	0.34^d^	0.10^c^			61.50^a^	26.51^d^	9.75^bc^	2.24^c^		
800	5.00	23.19^a^	20.11^a^	5.53^a^	2.52^a^			45.19^c^	39.34^a^	10.66^ab^	4.81^b^		
	0.50	16.55^b^	12.95^b^	4.22^b^	1.63^b^			46.51^c^	37.08^ab^	11.97^a^	4.45^b^		
	0.02	2.24^d^	1.04^d^	0.32^d^	0.02^c^			63.22^a^	27.54^d^	8.97^c^	0.27^d^		
ANOVA	K	<0.0001	<0.0001	<0.0001	<0.0001			<0.0001	<0.0001	0.0001	<0.0001		
	CO_2_	<0.0001	<0.0001	<0.0001	0.1877			0.6222	<0.0001	0.3046	<0.0001		
	K × CO_2_	0.0016	<0.0001	0.0091	0.8447			0.0741	0.0037	0.5475	0.2021		
**112 DAP**													
400	5.00	49.98^a^	28.4^b^	6.48^a^	121.62^b^	32.71^b^	88.91^b^	24.18^bc^	13.60^c^	3.11^b^	59.12^ab^	15.94^a^	43.17^ab^
	0.50	22.72^c^	11.53^d^	5.26^b^	59.53^d^	16.72^d^	42.80^d^	23.04^bc^	11.68^c^	5.37^a^	59.95^ab^	16.86^a^	43.08^ab^
	0.02	11.41^d^	3.34^e^	0.92^c^	3.24^e^	1.84^e^	1.40^e^	61.13^a^	17.10^b^	4.71^a^	17.06^c^	8.80^b^	8.26^c^
800	5.00	52.32^a^	42.39^a^	7.65^a^	157.52^a^	41.75^a^	115.77^a^	18.66^c^	16.65^b^	3.01^b^	61.68^a^	16.35^a^	45.33^a^
	0.50	40.71^b^	24.73^c^	6.78^a^	85.37^c^	23.58^c^	61.77^c^	24.67^b^	14.66^bc^	4.00^ab^	56.90^b^	15.42^a^	41.43^b^
	0.02	14.57^d^	5.53^e^	1.15^c^	3.08^e^	1.95^e^	1.13^e^	60.22^a^	23.03^a^	4.85^a^	11.90^d^	7.50^b^	4.39^d^
ANOVA	K	<0.0001	<0.0001	<0.0001	<0.0001	<0.0001	<0.0001	<0.0001	<0.0001	0.0038	<0.0001	<0.0001	<0.0001
	CO_2_	0.0421	<0.0001	0.0275	0.0211	<0.0001	<0.0001	0.1749	<0.0001	0.346	0.0891	0.1529	0.1689
	K × CO_2_	0.0681	<0.0001	0.4565	<0.0001	0.0021	0.0331	0.0485	0.2225	0.401	0.0107	0.2897	0.0075

† *The significance test (P-values) of the analysis of variance (ANOVA) between K and CO_2_ are given. Within columns, means followed by same letters are not significantly different at α = 0.05*.

*See Table 1 for other information*.

### Biomass partitioning was affected by K starvation and eCO_2_

Several plant organs showed a K × CO_2_ interaction attributed to the varying degree of K nutrition-dependent stimulation by eCO_2_ (Table 3). The dry weight of plant organs was significantly decreased with the decreasing K supply while eCO_2_ enhanced these traits, particularly under the two higher K levels (Table 3). Averaged across K treatments, the leaf, stem, root, and pod weight increased 27.6, 68.0, 23.0, and 33.4, respectively, under eCO_2_ at maturity. The K treatment significantly affected biomass partitioning among plant parts at all harvests. K starvation increased the average biomass partitioning to leaves (8–97%) across harvests and to the root fraction (54%) at maturity. However, partitioning to pods and seeds was drastically reduced, particularly under the 0.02 mM K treatment. CO_2_ treatment significantly affected partitioning to stems at 42 and 112 DAP. K × CO_2_ interaction was observed for biomass partitioning toward leaves, pods, and seeds at maturity. eCO_2_ increased the fraction of stems by 12.6–28.2% in the last two harvests when averaged across K levels. However, biomass partitioning toward pods and seed declined under eCO_2_ by 30.3% and 46.9%, respectively, under the 0.02 mM K treatment.

### K and N uptake and utilization efficiencies varied with K levels. the eCO_2_ stimulated KUE and NUE

KUpE and NUpE increased more than 4-fold between 28 DAP and plant maturity when averaged across treatments (Table 4). Averaged across CO_2_ levels, the K deficiency significantly decreased mean KUpE by 66.37% at 0.5 mM K and 80.4% at 0.02 mM K treatments across three harvests (Table 4). However, NUpE increased 6–72% under K deficiency when averaged across CO_2_ treatments at 29 and 42 DAP and remained unaffected at the maturity. KUE and NUE increased by 33.3- and 28.4-fold, respectively, with plant age between 28 DAP and maturity when averaged across treatments. Averaged across CO_2_ treatments, KUE increased by 42.8–124.2% under 0.5 mM K as compared with the control but decreased by 43.5–51.8% under 0.02 mM K treatment across harvests. However, compared with the control, NUE consistently declined by 48.2 and 93.2% at 0.5 and 0.02 mM K, respectively, when averaged across CO_2_ levels and harvest dates. eCO_2_ increased both KUE and NUE, especially under control (34.8–45.3%) and 0.5 mM K treatments (79–95%) across all harvests.

**Table 4:** Effect of CO_2_ (μmol mol^−1^) and potassium (K; mM) treatments on K and N uptake efficiency [KUpE and NUpE; mg K (g root dry weight)^−1^] and utilization efficiency [KUE and NUE; g^2^ dry weight (mg K)^−1^] of soybean plants at 28, 42, and 112 (or maturity) days after planting (DAP).

**CO_2_**	**K**	**KUpE**			**NUpE**			**KUE**			**NUE**		
		**28 DAP**	**42 DAP**	**112 DAP**	**28 DAP**	**42 DAP**	**112 DAP**	**28 DAP**	**42 DAP**	**112 DAP**	**28 DAP**	**42 DAP**	**112 DAP**
400	5.00	190.9^a^	250.8^b^	870.2^a^	273.6^b^	344.0^b^	1297.0	0.229^cd^	1.30^cd^	8.23^c^	0.159^b^	0.96^b^	5.47^b^
	0.50	68.0^b^	82.4^c^	366.2^b^	276.8^b^	416.2^b^	1060.6	0.293^bc^	2.36^b^	7.54^c^	0.071^c^	0.47^c^	2.34^d^
	0.02	50.9^bc^	54.0^d^	124.7^cd^	385.7^a^	677.6^a^	1482.3	0.141^e^	0.70^e^	4.36^d^	0.017^d^	0.07^*d*^	0.31^e^
800	5.00	190.6^a^	290.2^a^	832.1^a^	229.5^b^	349.7^b^	1377.1	0.315^b^	1.75^c^	10.90^b^	0.259^a^	1.47^a^	6.56^a^
	0.50	69.2^b^	67.7^cd^	266.6^bc^	236.9^b^	346.2^b^	1181.9	0.484^a^	4.48^a^	17.32^a^	0.136^b^	0.90^b^	3.60^c^
	0.02	45.0^c^	53.6^d^	107.6^d^	404.3^a^	629.2^a^	1405.5	0.166^*de*^	0.77^de^	5.82^d^	0.018^d^	0.07^d^	0.44^e^
ANOVA^†^	K	<0.0001	<0.0001	<0.0001	<0.0001	<0.0001	0.1318	<0.0001	<0.0001	<0.0001	<0.0001	<0.0001	<0.0001
	CO_2_	0.7795	0.2862	0.2012	0.2271	0.2069	0.7418	<0.0001	<0.0001	<0.0001	<0.0001	<0.0001	<0.0001
	K × CO_2_	0.8760	0.0141	0.7021	0.2835	0.0805	0.7897	0.0224	<0.0001	<0.0001	0.0246	0.0021	0.0025

† *The significance test (P-values) of the analysis of variance (ANOVA) between K and CO_2_ are given. Within columns, means followed by same letters are not significantly different at α = 0.05*.

*See Table 1 for other information*.

### Tissue K concentration was inversely related to N, N/K ratio, SLW, and root/shoot ratio while P_net_, DM, and seed yield increased with tissue K and showed greater response to eCO_2_

**Figure 3:**
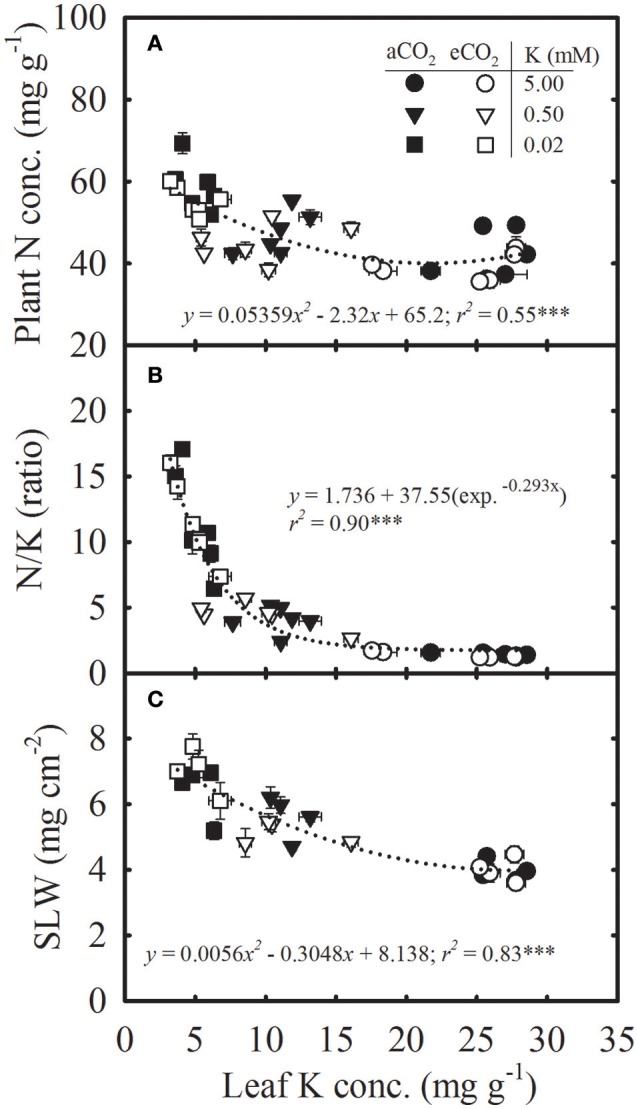
Relationship of leaf potassium (K) concentration with **(A)** plant nitrogen (N) concentration, **(B)** N/K ratio, and **(C)** specific leaf weight (SLW) of soybean grown under ambient (aCO_2_; filled symbols, 400 μmol mol^−1^) and elevated CO_2_ (eCO_2_; open symbols, 800 μmol mol^−1^) at three K levels. Symbols represent the mean ± SE of six individuals measured across the repeated experiment. Data are pooled from the destructive harvests at 28, 42, and 112 **(A,B)** or 28 and 42 **(C)** days after planting. Dotted lines represent either the polynomial second-order **(A,C)** or exponential decay **(B)** equation fitted across CO_2_ treatments. The significance level of the regression analysis for each equation is indicated as ^***^ representing *P* ≤ 0.001. Error bars smaller than the symbols are not visible.

Plant N concentration, N/K ratio, and SLW increased curvilinearly as the leaf K concentration decreased without a significant difference between CO_2_ treatments (Figure 3). Based on the regression fit, a decreased leaf K concentration from 28.5 to 3.2 mg g^−1^ led to an increase in plant N concentration from 42.5 to 58.2 mg g^−1^ (Figure 3A). The leaf N/K ratio increased exponentially exhibiting the greatest increase below roughly 10 mg g^−1^ leaf K concentration (Figure 3B).

**Figure 4:**
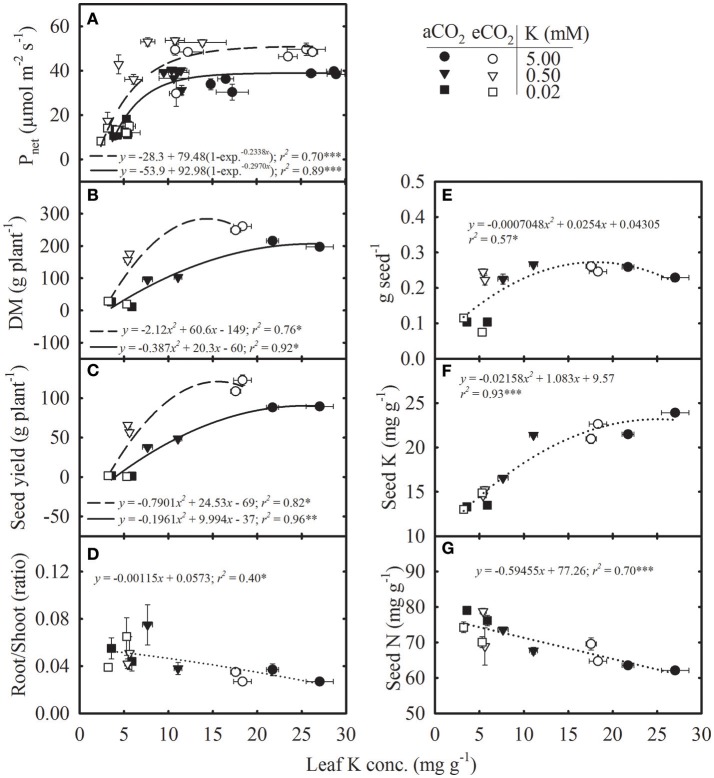
Relationship of leaf potassium (K) concentration with **(A)** photosynthesis (P_net_), **(B)** total dry matter (DM), **(C)** seed yield, **(D)** root/shoot ratio, (E) individual seed weight (g seed^−1^), (F) seed K concentration, and (G) seed nitrogen (N) concentration of soybean grown under ambient (aCO_2_; filled symbols/solid lines, 400 μmol mol^−1^) and elevated CO_2_ (eCO_2_; open symbols/dashed lines, 800 μmol mol^−1^) at three K levels. Symbols represent the mean ± SE of at least six individuals measured across the repeated experiment six times in the season in the uppermost fully expanded leaves **(A)** or from the destructive harvests at maturity **(B–G)**. Lines are the fit of either exponential rise to maximum-3 parameters **(A)**, polynomial second-order **(B,C,E,F)** or linear regression **(D,G)** equations. Dotted lines represent the fit across CO_2_ treatments. The significance level of the regression analysis for each equation is indicated as ^*^, ^**^, or ^***^ representing *P* ≤ 0.05, *P* ≤ 0.01, and *P* ≤ 0.001, respectively. Error bars smaller than the symbols are not visible.

The P_net_, DM, and seed yield increased non-linearly with leaf K concentration, and the response was greater under eCO_2_ (Figures 4A–C). The P_net_ appeared to be steady above ~8 mg g^−1^ leaf K concentration (Figure 4A). Root/shoot ratio exhibited an inverse linear relationship with leaf K concentration (Figure 4D). The individual seed weight and seed K concentration increased curvilinearly with leaf K concentration and did not differ between CO_2_ levels (Figures 4E,F). However, seed N concentration decreased with increase in leaf K concentration similarly across CO_2_ levels (Figure 4G).

## Discussion

### Potassium deficiency limited growth and yields more than photosynthetic processes

Despite the 54% decrease in K concentration in the uppermost fully expanded leaves under 0.5 mM vs. control (5 mM) K supply, the mean P_net_ did not decline at either CO_2_ level. In fact, other critical components of photosynthesis, such as ${\text{F}}_{\text{v}}^{\prime }$/${\text{F}}_{m}^{\prime }$, Φ_PSII_, q_P_, T, *g*_s_, C_i_, and TChl, also remained unaffected, indicating that the mean leaf K concentrations of between 10.4 and 23.0 mg g^−1^ at aCO_2_ and 9.1 and 20.0 mg g^−1^ at eCO_2_ were satisfactory for optimum photosynthetic processes. Similarly, a stable P_net_ across a wide range of leaf K concentrations (between roughly 12 and 40 mg g^−1^) was evident in cotton and alfalfa from the previous studies (Cooper et al., 1967; Reddy and Zhao, 2005). In contrast to the P_net_, the averaged TLA, total DM, and seed yields declined by ~45% under 0.5 mM vs. control K regardless of CO_2_ level. This was primarily attributed to the decreased plant height, leaf number, biomass accumulation, and number of pods per plant. However, under severe K deficiency (0.02 mM K), the P_net_, *g*_s_, and TChl exhibited about a 75% reduction as compared with the control, which was still less than the approximately 90% decreases observed for TLA, DM, and seed yield. The lower seed yield under severe K deficiency was associated with reduced pod and seed numbers and reduced seed size. Thus, severe K deficiency was required to induce a substantial decline in photosynthetic processes as compared with growth parameters, validating part of the hypothesis that “the growth and photosynthetic responses to K starvation would differ in soybean depending on the level of K supplied.” Previous studies also suggested that the rate of photosynthesis declined under severe K starvation, whereas biomass accumulation was sensitive to even mild K deficiency (Andrews and Svec, 1976; Gerardeaux et al., 2009).

Under severe K deficiency, a drastic reduction of photosynthetic processes was in agreement with other studies in soybean and cotton (Andrews and Svec, 1976; Reddy and Zhao, 2005; Gerardeaux et al., 2009; Li et al., 2011). The decline of *g*_s_ was accompanied by decreased T and C_i_ under severe K deficiency, which might indicate the partial contribution of stomatal limitation to overall photosynthetic capacity. However, previous studies indicated that biochemical limitations and reduced mesophyll conductance to CO_2_ might have a greater impact on the P_net_ limitations under K deficiency (Jin et al., 2011; Battie-Laclau et al., 2014; Lu et al., 2016b; Jákli et al., 2017). The beneficial effect of eCO_2_ on photosynthetic processes under control and 0.5 mM K treatment suggested that a steeper CO_2_ gradient might facilitate CO_2_ diffusion through stomata and mesophyll cells (Singh et al., 2013b). However, the lack of eCO_2_ stimulation on photosynthesis-related traits under severe K deficiency indicated that the P_net_ might be primarily limited by non-stomatal factors, such as mesophyll resistance to CO_2_ diffusion and the inhibition of the photo-biochemical processes. Reduced function of PSII photochemistry, mesophyll conductance, carboxylation capacity, rate of electron transport, chlorophyll biosynthesis, and assimilate transport due to decreased phloem loading have been reported under K deficiency in several species (Gerardeaux et al., 2009; Jin et al., 2011; Battie-Laclau et al., 2014; Lu et al., 2016b; Jákli et al., 2017). Moreover, the larger reduction in growth compared with the reduction in photosynthetic properties may also lead to the downregulation of photosynthetic potential due to limited sink capacity, which was also reflected by lower C/N ratios (Singh et al., 2013b). The stress-mediated photosynthetic limitations on the photo-biochemical turnover of the absorbed light energy [NADP(H), ATP] results into excess excitation of PSII, which may lead to the impairment of the PSII reaction center in the chloroplast (Marschner and Cakmak, 1989; Cakmak, 2005). In fact, reduced chlorophyll and impairment of the photochemical properties was also evident in this study by the concomitant reduction of the photochemical efficiency and quantum yield of PSII (e.g., ${\text{F}}_{\text{v}}^{\prime }$/${\text{F}}_{\text{m}}^{\prime }$ and Φ_PSII_) under severe K deficiency. The high Φ_PSII_/Φ_CO2_ ratio indicated consumption of electrons by sinks other than CO_2_ fixation (pseudocyclic electron flux, N metabolism, and photorespiration), supporting the observed lower quantum yield (Φ_PSII_) in severely K-deficient plants (Brooks, 1986; Edwards and Baker, 1993).

The regression relationships of the plant processes with tissue K concentration provided insight into the changes in their functional relationship across a gradient of leaf K at different CO_2_ levels (Figures 3, 4). The N accumulation in plants led to the inverse relationship observed between tissue K and N concentrations similarly across CO_2_ levels but resulted in reduced C/N and increased N/K ratios under K deficiency. The mean N/K ratio in the uppermost fully expanded leaves was below two under control conditions whereas it increased up to 16 in K-deficient plants, indicating K as the primary growth-limiting factor (Lawniczak et al., 2009). When growth is limited by other nutrients, the excess N might be stored in the form of nitrogenous compounds (e.g., non-functional protein, amino acids) at the expense of other cellular components such as chlorophyll and enzymes (Staswick et al., 1991; Singh et al., 2016). In the present study, the accumulation of N was exacerbated by the increase in N supply in the K- deficient treatments. The eCO_2_ modified the exponential relationship between P_net_ and leaf K, with a greater P_net_ observed above roughly 8 mg g^−1^ tissue K concentration. However, below this (8 mg g^−1^) leaf K concentration, P_net_ did not differ between CO_2_ levels and sharply declined. A similar reduction in photosynthesis with leaf K concentration has also been observed in other species (Reddy and Zhao, 2005; Jin et al., 2011). The DM and seed yield were also increased in response to increasing leaf K concentration, and productivity was always greater under eCO_2_ for a given leaf K concentration, except under very low leaf K. Moreover, as compared with the P_net_, the DM and seed yield began to decline at the higher level of leaf K concentration indicating greater sensitivity to K deficiency. For instance, the DM and seed yield declined while P_net_ remained unaffected roughly near leaf K concentration of 11 mg g^−1^ (Figures 4A–C). However, regression relationships for DM and seed yield should be treated with caution because of the large gap between leaf K concentrations of plants supplied with 5.0 vs. 0.5 mM K treatments. The eCO_2_ did not modify the relationship of root/shoot ratio, individual seed weight, or the concentration of seed K and N to the concentration of leaf K, which was in agreement with a previous study conducted under P nutrition in soybean (Singh et al., 2014). The inverse relationship between seed N and leaf K concentration suggested that N mobilization to the seed was not restricted and the seed N demand may be lower due to decreased seed yield under K deficiency (Singh et al., 2014).

### Under severe K starvation, biomass accumulation and partitioning tended to decrease or increase, respectively, for vegetative plant organs but declined consistently for reproductive organs

Biomass partitioning and the allocation of nutrients among plant organs are important traits that may reflect the plant ability to adjust physiological and metabolic processes under a given situation. The vegetative plant organs (leaves, stems, and roots) at maturity responded to the K starvation by decreased biomass accumulation but increased partitioning, especially under severe K deficiency. Interestingly, the biomass partitioning toward the reproductive organs (pod and seed yield) was largely unaffected between the control and 0.5 mM K supply (Table 3). However, under severe K deficiency, the partitioning to pod and seed yields decreased by over 75%. Thus, under 0.5 mM K supply, the decreased yield primarily resulted from a direct decline in yield-related traits, whereas under severe K deficiency, both a decline of and the reduced partitioning toward yield-related traits contributed to the yield loss. K deficiency has been shown to restrict fruit production to a greater extent than vegetative growth in cotton (Reddy and Zhao, 2005; Read et al., 2006). The biomass partitioning toward the leaves may promote more leaf area in an effort to enhance carbon assimilation and increase the carbohydrate supply. Increased biomass partitioning toward the roots under K deficiency was only observed at maturity that led to the inverse relationship between root/shoot ratio and leaf K concentration (Figure 4D). The large decline in the fraction of reproductive structures (e.g., pod and seed yield) under severe K deficiency may have increased the root/shoot ratio. In fact, no significant relationships (linear: *r*^2^ = 0.002, *P* = 0.9646; polynomial second order: *r*^2^ = 0.16, *P* = 0.4469, data not shown) between root/shoot ratio and leaf K concentration was observed when the pod weight was excluded in calculation of root/shoot ratio. Previous studies did not find a consistent increase in the root/shoot ratio in other crops under K-limited conditions (Cakmak et al., 1994; Reddy and Zhao, 2005; Lawniczak et al., 2009; Ma et al., 2013). The inhibition of sugar translocation has been suggested to suppress the biomass allocation to the roots under K deficiency (Cakmak et al., 1994; Römheld and Kirkby, 2010). However, an increased root/shoot ratio has often been reported under N- or P-limited conditions (Römheld and Kirkby, 2010; Lenka and Lal, 2012; Singh et al., 2013b, 2014).

eCO_2_ tended to stimulate dry matter production at maturity in all plant organs, except under severe K deficiency. The eCO_2_ did not affect the biomass partitioning to leaves and roots but enhanced the partitioning to stems at maturity across K levels. However, eCO_2_ decreased the partitioning to pods and seeds, especially under severe K deficiency. Thus, eCO_2_ appears to increase the sensitivity of the reproductive organs (e.g., pods and seed yield) to K deficiency, particularly under severe K starvation. Increased sensitivity to K deficiency under eCO_2_ has also been reported previously in cotton (Reddy and Zhao, 2005).

### Growth stimulation by eCO_2_ was greatest under 0.5 mM K followed by control K treatment, but was insignificant under severe K deficiency

Interestingly, the stimulation of TLA and DM under eCO_2_ vs. aCO_2_ was greater (60–80%) under the 0.5 mM K treatment than under the control K treatment (23–44%) across all harvests. Similarly, this stimulation of seed yield was 44 and 30% under 0.5 mM K and control K treatments, respectively. However, eCO_2_-mediated stimulation of these traits was insignificant under severe K deficiency (0.02 mM K). A similar observation can also be derived in cotton grown under varying K nutrition levels at 360 and 720 μmol CO_2_ from an earlier report by Reddy and Zhao (2005). However, unlike K deficiency, P deficiency consistently decreased the degree of stimulation by eCO_2_ with decreasing P supply (Cure et al., 1988; Singh et al., 2013a, 2014). For instance, Singh et al. (2013a, 2014) reported a consistent decrease in the degree of eCO_2_-mediated stimulation of photosynthesis, leaf area, and biomass production with decreasing P supply in soybean and cotton. In the current study, however, a consistent decline in the degree of P_net_ stimulation by eCO_2_ with decreasing K supply was also observed. In addition, the lack of soybean growth response to eCO_2_ under severe K deficiency was in agreement with the previous observations made under severe nutrient deficiency (Cure et al., 1988; Reddy and Zhao, 2005; Singh et al., 2013a, 2014)

One of the likely causes of greater eCO_2_-mediated growth stimulation at 0.5 mM K may be a lesser degree of decrease in growth parameters at eCO_2_ vs. aCO_2_ when K supply was reduced from control level to 0.5 mM K. For instance, at maturity, the decrease in DM and seed yields under 0.5 mM K vs. control K was 42–52% under aCO_2_ and 31–46% under eCO_2_. Nevertheless, soybean achieved the highest productivity under control K supply for each CO_2_ level. As compared with the control K treatment, the relative decline in growth parameters (e.g., P_net_, TLA, DM, and seed yield) under severe K deficiency was almost parallel (>90%) for a given CO_2_ level (aCO_2_ or eCO_2_). Thus, plants grown under a CO_2_-enriched environment might experience a similar decrease in growth due to severe K deficiency as those grown under ambient CO_2_. However, soybean grown under eCO_2_ appeared to benefit by enhancing P_net_ and overall growth, to some extent, as compared with plants grown under aCO_2_, particularly where K deficiency was not severe, indicating better utilization of tissue-available K (i.e., greater KUE). Thus, our hypothesis that depending on the severity of K deficiency eCO_2_ will compensate, at least partially, the adverse effect of K starvation on plant growth and yield was largely validated.

### KUE was enhanced under 0.5 mM K treatment but reduced under severe K deficiency, while eCO_2_ consistently improved KUE and NUE

The mean tissue K concentration of the uppermost fully expanded leaves under control K treatment (21.5 mg g^−1^) was comparable with those observed previously in soybean and other crops grown in fertilized fields or under controlled environments (Hanway and Weber, 1971; Andrews and Svec, 1976; Reddy and Zhao, 2005; Read et al., 2006). Despite a consistent decrease of KUpE with decreasing K supply, the averaged KUE was 65.68% greater at 0.5 mM vs. the control K treatment across the three harvests. This was in agreement with previous studies reporting higher P utilization efficiency of soybean plants grown under P starvation (Cure et al., 1988; Singh et al., 2014). Although, in the current study, the high KUE signified plant ability to better utilize tissue K concentration under 0.5 mM K supply than under control K levels, a loss of soybean growth still occurred. Nonetheless, it was apparent that the decrease in plant biomass was less than the reduction in tissue K concentration, which resulted in an increased KUE under 0.5 mM K treatment (Singh et al., 2015). In contrast, a substantial decline in the KUE under severe K deficiency may be attributable to the impairment of plant metabolic processes and a greater reduction of biomass accumulation than tissue K concentration (Gerardeaux et al., 2009; Singh et al., 2015). Impairment of the plant metabolic functions restricts plant growth and development when the tissue nutrient concentration falls below a threshold level (Siddiqi and Glass, 1981; Bednarz and Oosterhuis, 1999; Cakmak, 2005). Thus, a decline in the KUE under severe K deficiency was not surprising. An increased NUpE and tissue N accumulation led to a marked consistent reduction in the NUE in K-deficient plants. Lower NUE has also been previously reported in plants grown under P-deficient conditions (Singh et al., 2013a, 2014).

The soybean plants appeared to allocate the highest K concentration to the stems followed by the leaves. The K allocation to the leaves is critical for the photosynthetic processes, whereas nutrients allocated to the stems play a vital role in the internal nutrient recycling and transport mechanisms (Yan et al., 2016 and references therein). Since adequate K plays an important role in the phloem loading and assimilates transport, the observed high stem K concentration was not surprising (Huber, 1984; Cakmak, 2005).

Similar to the current study, lower concentrations of nutrient elements (e.g., K and N) under eCO_2_ have often been reported and might be attributed to the increased demand, lower transpiration, increased growth, and leaf thickness (Cure et al., 1988; Taub and Wang, 2008). In fact, greater SLW (an indicator of leaf thickness), lower *g*_s_ and transpiration, and increased soybean growth were observed for the plants grown under eCO_2_. The enhancement of KUE under eCO_2_ indicated that soybean plants produced greater biomass and seed yield with relatively lower tissue K concentration under eCO_2_ vs. aCO_2_; thus, exhibiting an efficient utilization of tissue-available K. This also partly explains the greater proportion of the eCO_2_-mediated stimulation of growth observed at 0.5 mM vs. the control K treatment. The lack of growth stimulation by eCO_2_ under severe K deficiency might have been caused by very low tissue K concentration, which failed to support increased growth under eCO_2_ as also found in another study (Reddy and Zhao, 2005).

### Increased SLW, and Φ_PSII_/Φ_*CO*2_ ratios but decreased q_P_, chlorophyll concentration, and overall plant size were some of the adaptation mechanisms under K deficiency

The increased SLW under K deficiency was in agreement with previous studies and might partly be attributed to the reduced cell expansion, accumulation of photosynthates due to limited translocation, and decreased plant size (Gerardeaux et al., 2009; Jin et al., 2011). The increased leaf thickness might also enhance the potential photosynthetic capacity of plant leaves (Garnier et al., 1999). A simultaneous decline of ${\text{F}}_{\text{v}}^{\prime }/{\text{F}}_{\text{m}}^{\prime }\Phi_{\text{PSII}}$, and q_P_ under severe K deficiency indicated reduced efficiency and quantum yield of PSII, photochemical quenching (*q*_P_), and interrupted electron transport chain (Singh and Reddy, 2015). The ${\text{F}}_{\text{v}}^{\prime }$/${\text{F}}_{\text{m}}^{\prime }$ has been found to be inversely related to non-photochemical quenching (Weis and Berry, 1987). However, the Φ_PSII_ value provides a direct estimate of the efficiency of light use for electron transport by PSII (Baker and Rosenqvist, 2004). Higher Φ_PSII_/Φ_CO2_ observed under severe K deficiency indicated the presence of an alternative electron sink including photorespiration to dissipate the excess energy of the PSII reaction center (Edwards and Baker, 1993; Jacob and Lawlor, 1993; Singh and Reddy, 2014, 2015). Lower leaf chlorophyll concentration can serve as a mechanism to avoid light absorption and to minimize plant sensitivity to light under K stress conditions (Cakmak, 2005; Singh and Reddy, 2016). Thus, the lower chlorophyll concentration and decreased Φ_PSII_ and q_P_ along with increased Φ_PSII_/Φ_CO2_ appeared to be some of the mechanisms employed to minimize light absorption and excitation pressures into the PSII reaction center under severe K deficiency (Singh and Reddy, 2016). The observed reduction of plant size (e.g., leaf area, biomass) under K-limited conditions might indicate a major strategy to reduce the overall nutrient demand and to support the allocation of carbon and energy toward the organ associated with acquiring the limited resources, such as leaves and roots (Bazzaz, 1997; Lenka and Lal, 2012).

## Conclusions

eCO_2_ showed the potential to enhance photosynthesis while reducing stomatal conductance under adequate K supply. This contributed to higher soybean productivity as represented by numerous growth and yield traits. However, under K deficiency, the beneficial effects of eCO_2_ were diminished. In addition, the K × CO_2_ interaction had a greater impact on the growth- and yield-related traits than on the photosynthesis-related parameters. Thus, K deficiency limited soybean growth traits more than photosynthetic processes regardless of the growth CO_2_ levels. The overall lower leaf area coupled with the retarded growth primarily contributed to the lower biomass and yield under K deficiency. Soybean plants adjusted to the K deficiency by decreasing plant size to reduce nutrient demands, increasing SLW, and biomass partitioning to leaves throughout the growing season and to the roots at maturity. In addition, reduced TChl concentration and photochemical quenching, and greater Φ_PSII_/Φ_CO2_ appeared to assist in reducing light absorption and the excess excitation pressure of PSII to minimize photoinhibition in severely K-deficient plants. Despite a consistent decline in the KUpE, the increased KUE indicated the plants' ability to better utilize the available tissue K, except under severe K deficiency. The degree of soybean growth stimulation by eCO_2_ was greater under 0.5 mM K than under the control K treatment, but such stimulation was not observed under severe K deficiency. Thus, the eCO_2_ compensated, at least partially, for decreases in soybean growth and seed yield depending upon the K nutrition. A severe K deficiency diminished the positive effects of eCO_2_ on P_net_ and total biomass production and completely suppressed the increased seed yield.

## Author contributions

SS and VR conceived the experiment. SS designed and conducted the experiment, collected and analyzed the data, and wrote the manuscript. Both authors approved the final manuscript.

### Conflict of interest statement

The authors declare that the research was conducted in the absence of any commercial or financial relationships that could be construed as a potential conflict of interest.
